# Weekly Oral Prophylaxis With MK-8527 Protects Rhesus Macaques From Intrarectal Challenge With Simian–Human Immunodeficiency Virus

**DOI:** 10.1093/infdis/jiaf610

**Published:** 2025-12-16

**Authors:** Tracy L Diamond, Yash Kapoor, Fangbiao Li, Bang-Lin Wan, Melissa A Boddicker, Jill W Maxwell, Winnie Ngo, Jane A Fontenot, Kelly A Soileau, Francois J Villinger, Jay A Grobler, Shubing Wang, Kerry L Fillgrove, Henry S Lange, Ernest Asante-Appiah

**Affiliations:** MRL, Merck & Co., Inc., Rahway, New Jersey, USA; MRL, Merck & Co., Inc., Rahway, New Jersey, USA; MRL, Merck & Co., Inc., Rahway, New Jersey, USA; MRL, Merck & Co., Inc., Rahway, New Jersey, USA; MRL, Merck & Co., Inc., Rahway, New Jersey, USA; MRL, Merck & Co., Inc., Rahway, New Jersey, USA; MRL, Merck & Co., Inc., Rahway, New Jersey, USA; New Iberia Research Center, University of Louisiana at Lafayette, New Iberia, Louisiana, USA; New Iberia Research Center, University of Louisiana at Lafayette, New Iberia, Louisiana, USA; New Iberia Research Center, University of Louisiana at Lafayette, New Iberia, Louisiana, USA; MRL, Merck & Co., Inc., Rahway, New Jersey, USA; MRL, Merck & Co., Inc., Rahway, New Jersey, USA; MRL, Merck & Co., Inc., Rahway, New Jersey, USA; MRL, Merck & Co., Inc., Rahway, New Jersey, USA; MRL, Merck & Co., Inc., Rahway, New Jersey, USA

**Keywords:** MK-8527, pre-exposure prophylaxis, simian–human immunodeficiency virus, HIV-1 prevention, nonhuman primate

## Abstract

**Background:**

MK-8527 is a novel nucleoside reverse transcriptase translocation inhibitor being evaluated for prevention of HIV-1 acquisition. MK-8527 is phosphorylated intracellularly to its active form, MK-8527-triphosphate, which inhibits HIV-1 replication. This study evaluated the efficacy of oral MK-8527 as pre-exposure prophylaxis in rhesus macaques challenged intrarectally with simian–human immunodeficiency virus (SHIV).

**Methods:**

Two groups of male macaques (*n* = 8 per group) received weekly oral doses of MK-8527 for 12 weeks. Starting 1 week after treatment initiation, the macaques received weekly intrarectal challenge with SHIV162P3 for 10 weeks. Each group was treated in 3 dosing panels: Group 1 received MK-8527 6, 1, and 0 mg/kg (vehicle only), whereas Group 2 received MK-8527 2, 0.3, and 0.1 mg/kg. A washout period of ≥4 weeks followed each dosing panel. A control group (*n* = 8) was challenged without receiving MK-8527. Plasma viral loads were monitored weekly, with infection confirmed by 2 consecutive measurements of SHIV RNA >100 copies/mL. Concentrations of MK-8527 phosphorylated forms were quantified using liquid chromatography-tandem mass spectrometry.

**Results:**

Once-weekly doses of MK-8527 ≥ 0.1 mg/kg for 12 weeks conferred complete protection against intrarectal SHIV acquisition, and 7/8 untreated control macaques and 5/8 vehicle-only macaques became infected. The rate of infection for macaques receiving MK-8527 was at least 11.1-fold (*P* = .009) lower compared with the control or vehicle group. MK-8527-triphosphate trough concentrations at 0.1 mg/kg resulted in a mean inhibitory quotient of 2.2.

**Conclusions:**

Prophylaxis with MK-8527 completely protected macaques from SHIV infection, supporting its further clinical development for prevention of HIV-1 acquisition.

## Teaser

MK-8527, a novel nucleoside reverse transcriptase translocation inhibitor, prevented SHIV acquisition in rhesus macaques at doses ≥0.1 mg/kg given weekly, demonstrating potential for low-dose, long-acting oral pre-exposure prophylaxis and supporting its clinical development for prevention of HIV-1 acquisition.

HIV-1 is a global public health concern; 1.3 million people were newly diagnosed in 2023 [1]. Pre-exposure prophylaxis (PrEP) has emerged as an effective strategy for preventing HIV acquisition among populations who may benefit from PrEP [2–4]. Approved PrEP options include daily oral emtricitabine (FTC) combined with tenofovir disoproxil fumarate (TDF); daily oral FTC with tenofovir alafenamide; the monthly dapivirine vaginal ring; injectable cabotegravir (CAB-LA), administered every 2 months [5]; and lenacapavir, a recently approved twice-yearly injectable [6].

The effectiveness of PrEP is closely linked to medication adherence [7]. When taken as prescribed, PrEP reduces the likelihood of acquiring HIV through sexual transmission by approximately 99% and through injection drug use by approximately 74% [8, 9]. However, adherence to daily oral PrEP remains a significant challenge. In studies of cisgender men who have sex with men, high PrEP adherence (>80%) reduced HIV acquisition by 86%, whereas lower adherence (<80%) led to only a 45% reduction [7]. In the VOICE study, which evaluated various PrEP options in 5029 cisgender women in Africa, none of the regimens significantly reduced HIV-1 acquisition; low adherence was identified as the primary reason for lack of effectiveness [10]. A global systematic review and meta-analysis reported that, within 6 months of starting PrEP, approximately 70% of users had suboptimal adherence or had discontinued [11]. Adherence issues often stem from the daily commitment required with oral medications, side effects, concerns about drug resistance, and the stigma associated with HIV treatment [12]. Discontinuation may result from similar factors, as well as logistical barriers such as maintaining consistent access to medication or healthcare services [11]. Injectable CAB-LA and lenacapavir require administration by healthcare providers every 2 or 6 months, respectively [13–16]. Acceptance of and accessibility to these PrEP agents may be limited by healthcare service delivery systems, required clinic visits, and injection site reactions [17, 18]. These findings underscore the urgent need for developing new long-acting oral prevention options to improve adherence.

MK-8527, a novel once-monthly nucleoside reverse transcriptase translocation inhibitor currently in Phase 3 clinical development for HIV-1 PrEP [19, 20], has shown potential to become a long-acting oral PrEP option [21–23]. MK-8527 is phosphorylated intracellularly to its active triphosphate (TP) form, MK-8527-TP [22]. MK-8527-TP is a potent inhibitor of HIV-1 replication with a long intracellular half-life (*t*_1/2_), making it suitable for extended-duration dosing [22]. Phase 1 studies in adults without HIV showed that participants receiving single (0.5–200 mg) or multiple (5–40 mg; once weekly [QW] for 3 weeks) oral doses of MK-8527 had an adverse event profile similar to that of placebo [24]. For people living with HIV who had not previously taken any antiretroviral agents, single doses of MK-8527 (0.5–10 mg) resulted in significant decreases in viral load (≥1.0 log_10_ copies/mL) over 7 days after the initial dose [21]. In a Phase 2 study in adults with low likelihood of HIV-1 exposure, the pharmacokinetic (PK) of MK-8527 and MK-8527-TP supported monthly oral dosing for PrEP [23].

Nonhuman primate models, particularly those evaluating infection of rhesus macaques with simian–human immunodeficiency virus (SHIV), are frequently used to assess the effectiveness of potential new HIV-1 therapies, including those for PrEP [25]. These intrarectal SHIV challenge models closely mimic the route of sexual acquisition of HIV in humans, making them highly relevant for assessing preventative strategies [25]. This study evaluated the effectiveness of oral MK-8527 QW as PrEP in a macaque model using SHIV intrarectal challenge.

## METHODS

### SHIV Model

SHIV encoding the SF162P3 envelope (SHIV162P3) was used for all studies. SHIV162P3 stock was produced and titrated for the rectal route at New Iberia Research Center (New Iberia, LA, USA).

### Antiviral Activity of MK-8527 Against SHIV In Vitro

Rhesus peripheral blood mononuclear cells (PBMCs) were activated with concanavalin A (5 µg/mL, 3 days), then resuspended in interleukin-2–supplemented growth medium (RPMI-1640 with 10% fetal bovine serum, 100 U/mL penicillin, and 100 µg/mL streptomycin). Cells (6× 10^6^) were infected with SHIV via spinoculation, followed by incubation overnight at 37°C/5% CO_2_. Infected cells were washed and resuspended in growth medium, and antiviral activity was tested in PBMCs from 3 donors using 8- to 10-point, 3-fold serial dilutions of MK-8527, with final concentrations from 60 to 0.03 nM and 1% dimethyl sulfoxide (DMSO) in each well. Compound or DMSO control (without compound) was added to infected cells in 96-well plates and incubated at 37°C/5% CO_2_ for 3 days. Cells were then lysed with Triton X-100. Viral capsid (p27) was quantified using the SIVp27 antigen capture assay kit (Advanced Bioscience Laboratories). Data were background-subtracted and normalized to DMSO controls. Half-maximal inhibitory concentration (IC_50_) was determined by nonlinear 4-parameter curve fitting of the data; results were presented as geometric mean and standard deviation.

To determine the intracellular concentration of MK-8527-TP required for antiviral activity, activated PBMCs were treated for 24 hours with 4 nM of MK-8527, a concentration 10-fold higher than the in vitro IC_50_ determined in 4 independent experiments using PBMCs from 3 animals. Cell pellets were resuspended in 70% methanol/30% distilled water and stored at –80°C for determination of MK-8527-TP concentration by liquid chromatography-tandem mass spectrometry (LC-MS/MS). The MK-8527-TP concentrations were divided by 10 to extrapolate to the IC_50_, assuming a linear correlation between MK-8527 dose and MK-8527-TP concentrations [22].

### Study Design

**Figure 1.**
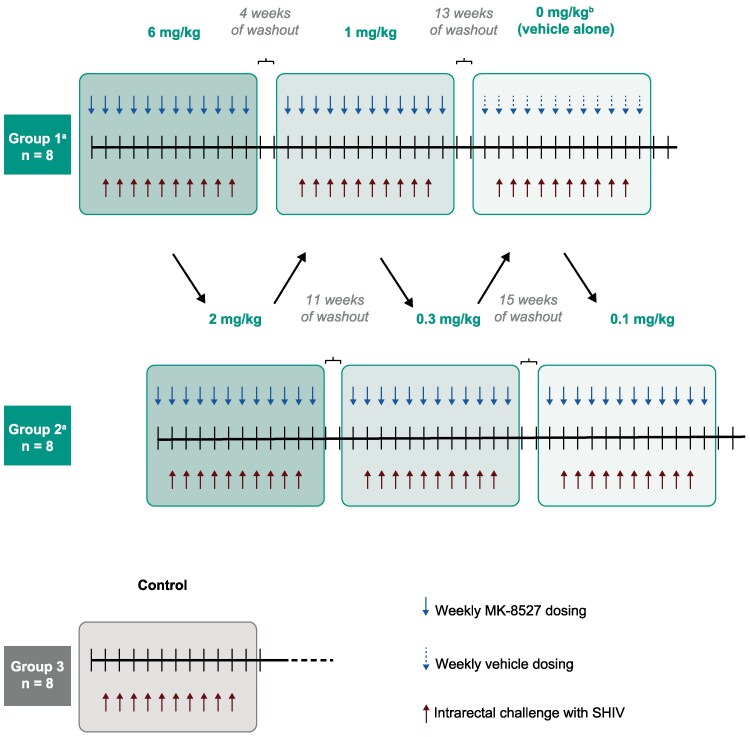
Study design of the intrarectal challenge model in male macaques. Arrows indicate the order of dosing. ^a^Uninfected animals were rechallenged and administered MK-8527 at a lower dose after ≥4 wks of drug washout. ^b^The 0 mg/kg (vehicle alone) dosing was included for Group 1 to verify that animals maintained susceptibility to SHIV infection after 20 challenges. SHIV, simian–human immunodeficiency virus.

Twenty-four male macaques, 9–11 years old, were divided into 3 groups of 8: two treatment groups (Groups 1 and 2) and 1 control group (Group 3). For each dosing level, Groups 1 and 2 received QW MK-8527 by oral gavage in 10% (w/v) polysorbate 80 vehicles for 12 consecutive weeks starting at Week 0. MK-8527 dosing levels proceeded from the highest to the lowest and alternated between the 2 treatment groups (Figure 1). Group 1 received 6, 1, and 0 mg/kg (vehicle only), whereas Group 2 received 2, 0.3, and 0.1 mg/kg. This alternating dosing strategy allowed for results from each dose panel to inform the dose selection for the other group.

Starting 1 week after treatment initiation for each panel, the animals received a weekly intrarectal challenge with SHIV162P3 (approximately 3× 50% animal infectious dose) for 10 consecutive weeks. Dosing and challenge were done within an hour at Weeks 1–10 to minimize the number of sedation cycles for the study animals. The untreated control group received the same viral challenges starting at Week 1 without MK-8527 prophylaxis. Between dosing panels in Groups 1 and 2, a period of ≥4 weeks was included for compound washout. Longer intervals between dosing panels allowed sufficient time for data analysis to inform on the next dosing level. Uninfected animals were administered new doses of MK-8527 and rechallenged as part of subsequent dosing panels (Figure 1).

Phlebotomy was performed for plasma viral load testing and measurement of MK-8527 concentrations in plasma, MK-8527-TP concentrations in PBMCs, and cell-associated viral DNA in PBMCs. Rectal biopsy specimens (≤20 approximately 2-mm-wide pinch biopsy samples of the colon mucosa) were collected at Week 12 (168 hours after the last MK-8527 dose) to measure MK-8527-TP concentrations.

### Plasma Viral Load Testing

Plasma viral load was monitored weekly for each dosing panel and during the washout periods using a quantitative real-time polymerase chain reaction (RT-PCR) assay and a standard curve of serially diluted simian immunodeficiency virus gag RNA of known concentration. Briefly, viral RNA was isolated using the QIAamp Viral RNA Mini kit (Qiagen) and quantitative RT-PCR was conducted using the Taqman RT-PCR Mix (2×) (Thermo Fisher Scientific) using primers/probes within the gag coding region. Viral load was quantified using 2 methods. Method 1, with a lower limit of quantification (LLOQ) of 100 copies/mL, used 140 µL of plasma, whereas Method 2, an enhanced viral load assay with a LLOQ of 10 copies/mL, used 1.4 mL of plasma and incorporated centrifugation at 21 000×g to concentrate the virus prior to isolating viral RNA. Method 2 was used at select time points to verify results from Method 1. Confirmation of infection required 2 consecutive viral loads exceeding 100 copies/mL.

### Quantification of Cell-Associated Viral DNA

To confirm that no infections occurred below the LLOQ of the viral load assay, cell-associated viral DNA from PBMCs was quantified by RT-PCR using a qualitative assay. Viral DNA was assessed in PBMCs obtained from animals after each panel of dosing/challenging was completed. Genomic DNA was isolated using the PureLink Genomic DNA Mini Kit (Thermo Fisher Scientific, MA, USA) and the presence of viral gag sequences was evaluated by amplification on the QuantStudio 12K Flex Real Time PCR System (Thermo Fisher Scientific, MA, USA). Samples with a threshold cycle (Ct) <40 were considered to be from infected animals (Ct values ranged from 29 to 38), whereas those with undetermined Ct were considered uninfected.

### Measurement of Phosphorylated MK-8527 in PBMCs

Two methods were developed for the analysis of MK-8527 monophosphate (MP), MK-8527 diphosphate (DP), and MK-8527-TP levels in PBMC samples stabilized with 70% methanol and stored at −70°C. In the first method, a 50-µL aliquot of each PBMC sample (thawed on ice) was treated with 50 µL of a methanol/0.1 M EDTA solution, whereas in the second method, samples were treated with 50 µL of 10% trichloroacetic acid. Both methods involved vigorous mixing, followed by centrifugation at 3220×g for 10 minutes, after which the supernatant was transferred to a clean 96-well plate for LC-MS/MS analysis.

While the concentration of analyte from LC-MS/MS is determined as molar concentration, concentrations of MK-8527-TP in PBMCs were converted to picomoles per 10^6^ cells by multiplying the measured MK-8527-TP concentration by sample lysate volume and then dividing by the total number of viable cells in the sample. While the LC-MS/MS assay had a LLOQ of 0.0005 µM for MK-8527-TP, the requirement to normalize the data based on the number of cells collected resulted in varying LLOQ for concentration reported in picomoles per 10^6^ cells. At lower dose levels, most PK samples collected at trough (168 hours after dosing) had concentrations < LLOQ for MK-8527-TP.

The *t*_1/2_ of MK-8527-TP was estimated using the measurable concentrations at 24 and 168 hours following administration across 5 dose levels (0.1, 0.3, 1, 2, and 6 mg/kg) after ≥3 doses. The MK-8527-TP *t*_1/2_ and concentration at 24 hours was used to extrapolate trough concentrations (*C*_trough_) of MK-8527-TP. The inhibitory quotient (IQ; *C*_trough_/IC_50_) was calculated for each extrapolated or observed *C*_trough_ value using the in vitro IC_50_ for MK-8527-TP in PBMCs. Arithmetic mean and standard deviation for the combination of extrapolated and observed IQ values were calculated.

### PK Study in Female Macaques

MK-8527 PK was evaluated in female macaques 11–14 years old (*n* = 4 per group) administered 3 oral doses of MK-8527 2 or 6 mg/kg QW using the same methods used for the male macaques. PBMCs, rectal tissue (≤20 pinch biopsies per sample), and vaginal tissue (≤5 approximately 5-mm-wide pinch biopsy samples per time point) were collected for analysis of MK-8527-MP, MK-8527-DP, and MK-8527-TP at 168 hours after the third dose.

### Measurement of Phosphorylated MK-8527 in Rectal and Vaginal Biopsies

Rectal and vaginal biopsies were cryomilled using a SPEX SamplePrep Freezer/Mill (SPEX SamplePrep LLC, Metuchen, NJ, USA), and tissue homogenates were prepared by adding 7 mL of a methanol/EGTA (70:30) solution per gram of cryomilled tissue. A 100-µL aliquot of each homogenate (thawed on ice) was treated with 100 µL of 10% trichloroacetic acid, followed by vigorous mixing and centrifugation at 3220×g for 10 minutes. The supernatant was transferred to a 96-well plate for LC-MS/MS analysis for MK-8527-MP, -DP, and -TP.

### Animal Welfare

Before the study, animals underwent a comprehensive physical examination, including a complete blood count, a comprehensive chemistry profile, and additional diagnostics as deemed necessary by the study veterinarian. Animals were single-housed indoors in appropriately sized cages for the study duration. After treatment initiation, daily observations were conducted to monitor for abnormal clinical signs, behavioral deviations, or signs of distress. Throughout the study, regular health monitoring included a serum chemistry panel and whole blood hematologic assessments.

The Institutional Animal Care and Use Committee (IACUC) at both Merck & Co., Inc., Rahway, NJ, USA, and the University of Louisiana at Lafayette reviewed and approved the care and use of animals, which were conducted in accordance with the principles outlined in the guidance of the Association for Assessment and Accreditation of Laboratory Animal Care, the Animal Welfare Act, the American Veterinary Medical Association Euthanasia Panel on Euthanasia, and the Institute for Laboratory Animal Research Guide to the Care and Use of Laboratory Animals.

### Statistical Analysis

The log-rank test was used to calculate differences between MK-8527 treated macaques and control animals at each dose level using GraphPad Prism v8 (Boston, MA, USA). The hazard ratios were estimated using the log-rank model.

## RESULTS

### MK-8527 Antiviral Activity Against SHIV

The antiviral activity of MK-8527 against SHIV162P3 was evaluated by monitoring p27 production in activated PBMCs. MK-8527 showed an IC_50_ of 0.40 ± 0.23 nM (*n* = 3) against SHIV, indicating potent antiviral activity. The geometric mean intracellular concentration of MK-8527-TP at the IC_50_ from 4 replicate experiments using activated PBMCs from 3 animals was 0.0063 ± 0.0032 pmol/10^6^ cells, equivalent to 0.0315 µM (Supplementary Table 1).

### Evaluation of Viremia in the SHIV Intrarectal Challenge PrEP Model

**Figure 2.**
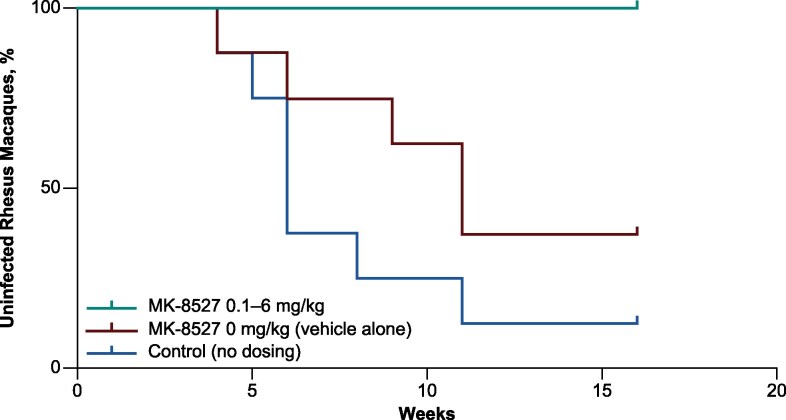
Infection status of macaques challenged with SHIV. Kaplan–Meir plot of macaques treated with MK-8527 0.1, 0.3, 1, 2, and 6 mg/kg and macaques remaining uninfected after serial SHIV challenges. Macaques were considered infected when 2 consecutive SHIV RNA values exceeded 100 copies/mL. SHIV, simian–human immunodeficiency virus.

To assess its use as prophylaxis in the SHIV challenge model, several dose levels of MK-8527 (0.1–6 mg/kg/week) were evaluated and compared with challenged untreated controls. Seven of 8 macaques (87.5%) in the untreated control group became infected after 3–10 intrarectal challenges with SHIV (Figure 2), confirming effectiveness of the viral challenge. Analysis of cell-associated proviral DNA in PBMCs collected from these animals confirmed the presence of viral DNA in infected animals and the absence of proviral DNA in the 1 uninfected animal.

Among macaques who received MK-8527 (0.1–6 mg/kg), no confirmed infections were observed after 10 SHIV challenges, based on the absence of plasma viremia and detectable proviral DNA in circulating PBMCs (Figure 2). Each panel that received MK-8527 ≥ 0.1 mg/kg showed an 18-fold (95% CI, 3.6–90.2) lower risk of SHIV infection than the untreated control group (*P* = .0004, by log-rank test).

To verify that the vehicle or previous exposures to SHIV that Group 1 and Group 2 had already received did not provide protection, the last panel for Group 1 was dosed with 10% (w/v) polysorbate 80 vehicle without MK-8527 (0 mg/kg, vehicle panel). Five of 8 macaques (62.5%) in this panel became infected after 3–10 SHIV challenges. The risk for SHIV infection did not differ between the untreated control and vehicle-only groups (*P* = .1457, by log-rank test). Analysis of cell-associated viral DNA in PBMCs from the vehicle-only animals confirmed the presence/absence of infection, similar to the untreated control group. This result shows that these animals and the untreated control group were similarly susceptible to infection. There was an 11.1-fold (95% CI, 1.8–67.7) lower risk for SHIV infection for animals in each panel that received MK-8527 ≥ 0.1 mg/kg compared with the panel that received vehicle only (*P* = .009, by log-rank test).

### PK and Tissue Distribution of MK-8527-TP

**Figure 3.**
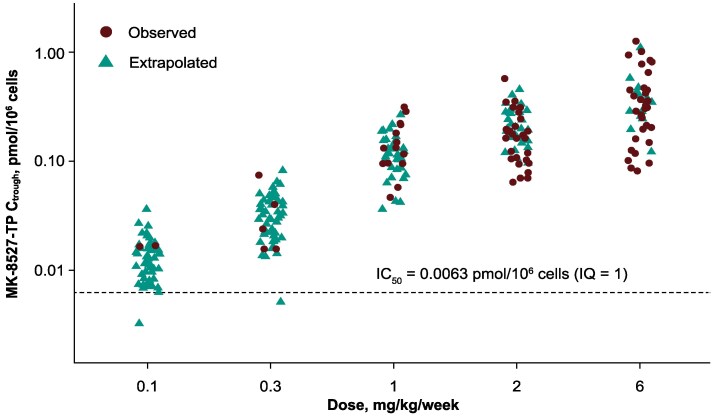
Observed and extrapolated MK-8527-TP *C*_trough_ in PBMCs at weekly doses of 0.1, 0.3, 1, 2, and 6 mg/kg. Observed: actual concentration values from collected PBMCs. Extrapolated: based on the extrapolation of observed C_24_ data and the *t*_1/2_ of MK-8527-TP in monkeys (39.9 h). C_24_, concentration 24 h after dosing; *C*_trough_, trough concentration; IC_50_, half-maximal inhibitory concentration; IQ, inhibitory quotient (*C*_trough_/IC_50_); MK-8527-TP, MK-8527 triphosphate; PBMC, peripheral blood mononuclear cell.

To evaluate the concentration of MK-8527-TP required for efficacy in the SHIV model, the *C*_trough_ of MK-8527-TP in PBMCs and rectal tissues was assessed in challenged animals. At lower doses of weekly MK-8527, MK-8527-TP concentrations at 168 hours after dosing were < LLOQ; therefore, additional PK collections were performed 24 hours after dosing to enable extrapolation of trough PK for MK-8527-TP at the lower dose levels (0.1 and 0.3 mg/kg). Utilizing data available at 24 hours and at 168 hours after dosing at the 5 different dose levels (0.1, 0.3, 1, 2, and 6 mg/kg), the MK-8527-TP *t*_1/2_ was determined to be 39.9 ± 10.6 hours (*n* = 54). The concentration at 24 hours and the *t*_1/2_ of MK-8527-TP was used to extrapolate and determine the MK-8527-TP concentration at 168 hours. The observed and extrapolated values for MK-8527-TP *C*_trough_ fell within the same range (Figure 3), supporting the concentrations extrapolated at the lower dose levels (0.1 and 0.3 mg/kg). The MK-8527-TP *C*_trough_ associated with the 0.1 mg/kg dose corresponded to a mean IQ of 2.2 (range, 0.5–5.8; Table 1).

**Table 1.** Mean Inhibitory Quotients based on Observed and Extrapolated MK-8527-TP *C*_trough_ in PBMCs From Macaques Receiving Each Dose of MK-8527 QW

MK-8527 Dose	Mean IQ ± SD	IQ Range
6 mg/kg	62.7 ± 44.2 (*n* = 48)	13.0–201.2
2 mg/kg	33.2 ± 16.1 (*n* = 60)	10.3–91.2
1 mg/kg	20.9 ± 10.1 (*n* = 47)	5.8–50.3
0.3 mg/kg	5.3 ± 2.6 (*n* = 53)	0.8–13.1
0.1 mg/kg	2.2 ± 1.0 (*n* = 50)	0.5–5.8

Abbreviations: *C*_trough_, trough concentration; IC_50_, half-maximal inhibitory concentration; IQ, inhibitory quotient (*C*_trough_/IC_50_); n, number of IQ data points; PBMC, peripheral blood mononuclear cell; QW, once weekly; MK-8527-TP, MK-8527 triphosphate.

Analysis of MK-8527-TP in rectal biopsies was performed on samples obtained 7 days (168 hours) after the last dose (dose 12) in each panel. The mean (SD) concentrations of MK-8527-TP in rectal tissue at trough levels for the 6, 2, and 1 mg/kg dosing panels were 0.0505 (0.0530) µM, 0.0171 (0.0177) µM, and 0.0096 (0.0051) µM, respectively. In the 1 mg/kg dosing panel, 3 of 8 animals had trough MK-8527-TP concentrations < LLOQ (0.004 µM). In the 0.3 and 0.1 mg/kg dosing panels, all trough rectal MK-8527-TP concentrations were < LLOQ. All animals in the 1 mg/kg dosing panel exhibited trough rectal MK-8527-TP concentrations below the in vitro IC_50_ of 0.0315 µM, indicating an IQ of <1 in rectal tissue for doses of ≤1 mg/kg.

### Evaluation of PK Exposure in Vaginal Tissue

**Figure 4.**
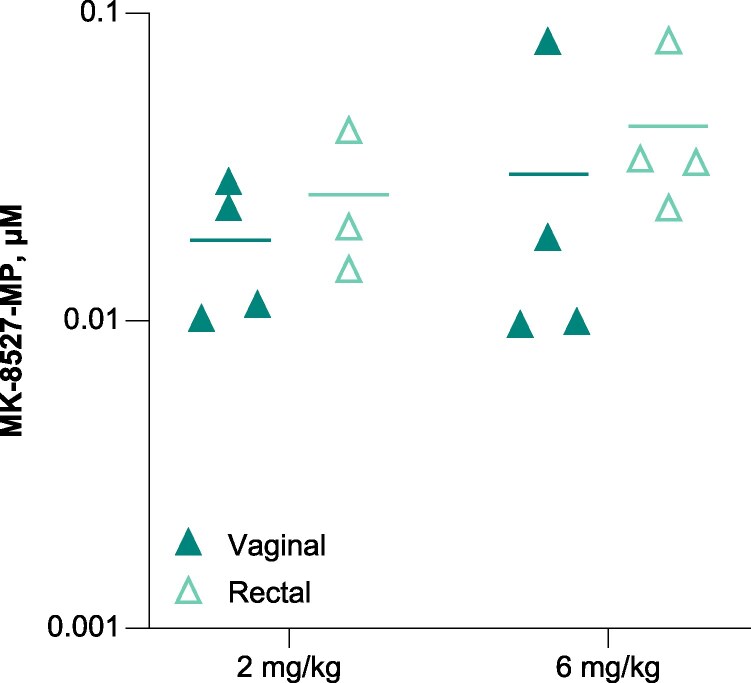
MK-8527-MP levels in vaginal and rectal tissue from female macaques. Scatter plot displays the concentration of MK-8527-MP at 168 h after the third weekly dose of MK-8527; horizontal lines depict the mean values. MK-8527-MP, MK-8527 monophosphate.

To compare MK-8527 exposure between female and male macaques, a PK study was conducted in female macaques (*n* = 4 per group). MK-8527-MP, -DP, and -TP levels in rectal biopsies in female macaques were similar to those observed in male macaques (data not shown). Because the amount of tissue obtained from vaginal biopsies were 3- to 7-fold lower than those obtained from rectal biopsies (due to differences in IACUC collection guidelines between the 2 tissue types), the amount of MK-8527-TP measured in the vaginal biopsies were highly variable. As the MK-8527-MP levels were about 2-fold higher than MK-8527-TP in both the rectal and vaginal tissue, MK-8527-MP was used as a surrogate to compare tissue levels of MK-8527 drug-related material. The levels of MK-8527-MP were comparable between vaginal and rectal biopsies in female macaques (Figure 4).

## DISCUSSION

MK-8527 given as prophylaxis provided complete protection against SHIV infection in a macaque rectal challenge model across all dosage groups, including the lowest dose of 0.1 mg/kg. Conversely, 87.5% of the untreated control group and 62.5% of the vehicle-only group became infected, confirming protection was derived from MK-8527 and not the vehicle. MK-8527 provided significant 18-fold and 11.1-fold reductions in SHIV acquisition risk compared with untreated control (*P* = .0004) and vehicle-only (*P* = .009) groups, respectively. Efficacy at the lowest dose (0.1 mg/kg) suggest a potential for low-dose, long-acting oral PrEP. Even at doses where MK-8527-TP concentrations in rectal tissue were below the in vitro IC_50_ (≤1 mg/kg), the drug conferred high protection against SHIV acquisition. These low MK-8527-TP concentrations may underestimate what is present in the target cells within the tissue due to composition and collection limitations of the biopsies. Similar levels of MK-8527 phosphorylated forms in rectal tissue from male and female macaques and in vaginal tissue from female macaques suggest that MK-8527 would likely perform similarly in a vaginal challenge study.

MK-8527 demonstrated an IC_50_ value of 0.40 ± 0.23 nM (*n* = 3) against SHIV162P3 comparable to the previously reported IC_50_ of 0.21 ± 0.09 nM (*n* = 93) against HIV-1 in human PBMCs [22]. The intracellular concentration of MK-8527-TP at the in vitro IC_50_ was also similar between macaques and humans (0.0063 pmol/10^6^ cells vs 0.0092 pmol/10^6^ cells) [22]. These findings suggest a comparable inhibitory potency of MK-8527 for SHIV and HIV-1 infections in PBMCs. The *t*_1/2_ of MK-8527-TP in humans is approximately 200 hours [24], approximately 5-fold higher than in macaques (39.9 hours). Learnings from the weekly challenge studies in the macaques can offer supporting evidence for the potential use and effectiveness of monthly MK-8527 in humans for PrEP.

The efficacy of MK-8527 in this study compares favorably to current FDA-approved PrEP options [26]. The estimated intracellular tenofovir DP concentration required for a 90% reduction in SHIV acquisition was 22.6 fmol/10^6^ PBMCs (IQ = 0.5) in male macaques treated with oral TDF/FTC [27] and 16 fmol/10^6^ PBMCs (IQ = 0.4) in male participants receiving once-daily oral TDF/FTC in the iPrEx study [28]. Although direct comparison between animal and human studies should be made cautiously, these results suggest that MK-8527 may offer similar or superior efficacy to current PrEP options, with the added benefit of a less-frequent dosing schedule that could significantly improve adherence and real-world effectiveness. The only long-acting prophylaxis options available that might benefit those struggling with adherence are CAB-LA and lenacapavir, which are administered by a healthcare provider every 2 or 6 months, respectively [13–16]. Although CAB-LA offers an alternative to oral PrEP, achieving sufficient concentrations for optimal protection in tissues remains a challenge [29, 30]. Oral administration of MK-8527 could mitigate complications associated with injectable PrEP, such as injection site reactions and implementation barriers within healthcare systems [9, 31].

The use of macaques with SHIV challenge closely mimics human HIV transmission [25]. The alternating design enabled dose optimization as the study progressed and minimized interanimal variability, providing a thorough PK analysis in target cells and tissue.

The PK properties of MK-8527-TP indicate that MK-8527 could be a viable candidate for less-frequent oral dosing schedules and support its evaluation as a monthly oral PrEP option in clinical studies [19, 20]. MK-8527 may help address adherence and persistence challenges associated with oral daily PrEP options, such as forgetfulness, stigma, and side effects [32]. The IC_50_ and intracellular concentrations of MK-8527-TP in PBMCs closely matched those from human PBMCs, reinforcing the relevance of the macaque model for predicting human efficacy. MK-8527 doses ≥0.1 mg/kg were effective in preventing SHIV acquisition in macaques. These findings support further clinical development of MK-8527 for the prevention of HIV-1 acquisition.

## Supplementary Material

jiaf610_Supplementary_Data

## Supplementary Data

Supplementary materials are available at *The Journal of Infectious Diseases* online (http://jid.oxfordjournals.org/). Supplementary materials consist of data provided by the author that are published to benefit the reader. The posted materials are not copyedited. The contents of all supplementary data are the sole responsibility of the authors. Questions or messages regarding errors should be addressed to the author.
