# Supplementary material for: Weekly Oral Prophylaxis With MK-8527 Protects Rhesus Macaques From Intrarectal Challenge With Simian–Human Immunodeficiency Virus
Source: J Infect Dis. 2025 Dec 16;233(4):e923–31. doi: 10.1093/infdis/jiaf610 (PMC13127747; doi:10.1093/infdis/jiaf610)
Supplement: jiaf610_Supplementary_Data [file jiaf610_supplementary_data.docx]

**Supplemental Material**

**Supplemental Table S1.**Intracellular Concentrations of MK-8527-TP at the IC_50_ in PBMCs From 4 Replicate Experiments, Using PBMCs From 3 Animals

| **Animal ID** | **MK-8527 nM** | **MK-8527-TP pmol/10^6^ cells** | **Extrapolate to IC_50_  of 0.4 nM by dividing by 10 pmol/10^6^ cells** |
| --- | --- | --- | --- |
| A17199 +  AK5G mixture | 4 | 0.067 | 0.0067 |
| A17L199 | 4 | 0.113 | 0.0113 |
| A16L168 | 4 | 0.048 | 0.0048 |
| A16L188 | 4 | 0.043 | 0.0043 |
|  |  | **Geometric mean** | 0.0063 |
|  |  | **Standard deviation** | 0.0032 |

Abbreviations: IC_50_, half-maximal inhibitory concentration; ID, identification; PBMC, peripheral blood mononuclear cell; TP, triphosphate.
